# Enzyme Reset: Water-Mediated Tautomerization Restores the Catalytic Asparagine in Protein *O*‑Fucosyltransferase 1

**DOI:** 10.1021/acs.jcim.6c00350

**Published:** 2026-04-10

**Authors:** Òscar Vidal-Gironès, Enrico Trizio, Peilin Kang, Michele Parrinello, José Pablo Rivas-Fernández, Carme Rovira

**Affiliations:** † Departament de Química Inorgànica i Orgànica (Secció de Química Orgànica) and Institut de Química Teòrica i Computacional (IQTCUB), 16724 Universitat de Barcelona, Martí i Franquès 1, Barcelona 08028, Spain; ‡ Atomistic Simulations, Istituto Italiano di Tecnologia, Via Enrico Melen 83, Genoa 16142, Italy; § Institució Catalana de Recerca i Estudis Avançats (ICREA). Passeig Lluís Companys 23, Barcelona 08010, Spain

## Abstract

Protein *O*-fucosyltransferase 1 (POFUT1) catalyzes the transfer of fucose to threonine or serine residues within epidermal growth factor-like domains (EGF-LDs) and is a therapeutic target for Notch-associated O-glycosylation disorders. Unlike classical inverting glycosyltransferases, POFUT1 employs a catalytic asparagine that tautomerizes to its imidic acid form during the reaction. How the enzyme subsequently restores the canonical amidic form of Asn51 has remained unclear. Here, quantum mechanics/molecular mechanics simulations reveal that active-site water molecules mediate Asn51 retautomerization through a Grotthuss-type proton relay involving a low free energy barrier (<6 kcal·mol^–1^). This process can occur regardless of the presence of product molecules in the active site, although it is most favorable after product release. These findings elucidate how POFUT1 resets its catalytic machinery after turnover, underscore the essential role of water molecules in enzyme catalysis, and suggest that similar water-mediated strategies may operate in other enzymes in which catalytic residues undergo protonation changes during turnover.

## Graphical abstract

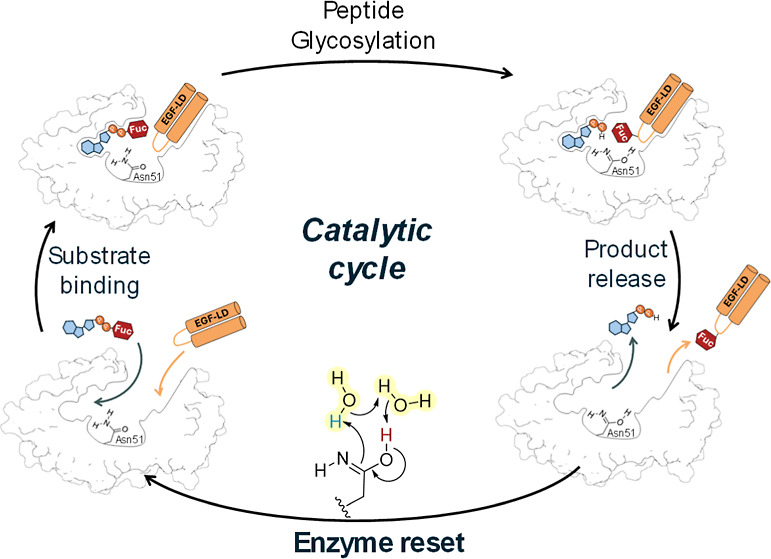

## 1 Introduction

Protein *O*-fucosyltransferase 1 (POFUT1) catalyzes the direct attachment of l-fucose to Ser/Thr residues, an important post-translational modification in higher eukaryotic organisms.^1^ The enzyme is particularly interesting for its involvement in the Notch signaling pathway (NSP), an essential cell–cell communication pathway conserved throughout the animal kingdom.^2,3^ This has been linked to several diseases in humans, such as the Dowling–Degos disease,^4^ leukemia,^5^ and colorectal cancer.^6^ POFUT1 is involved in one of the first steps of the Notch receptors’ maturation,^7,8^ being a potential therapeutic target for the aforementioned Notch-related disorders.

POFUT1 is a glycosyltransferase (GT) that catalyzes the transfer of l-fucose from guanosine diphosphate-β-l-fucose (hereafter fucose and GDP-Fuc, respectively) to the hydroxyl group of Ser or Thr with inversion of configuration at the anomeric carbon.^9^ The canonical mechanism for inverting GTs consists of a single-displacement S_N_2 reaction in which the acceptor nucleophile is deprotonated by a general base, typically Asp or Glu. However, structures of POFUT1 have shown that the active site contains an Asn residue, conserved among species, rather than the typical Asp or Glu.^9,10^ QM/MM simulations showed that, in spite of the low basicity of the carbonyl group of the Asn side chain, it can still act as a general base, as it bridges the acceptor Thr with the β-phosphate of the GDP-Fuc donor (Figure 1A).^11^ During the catalytic process, Asn51 shuttles a proton between the acceptor and the donor β-phosphate. As a consequence, Asn51 adopts the imidic acid tautomeric form, R–C­(=NH)­OH, after the chemical reaction. Importantly, enzymatic catalysis is inherently cyclic: after each turnover, the enzyme must return to the resting state to sustain multiple catalytic events. This recovery step is often not analyzed, as it is implicitly assumed to occur without energetic or structural consequences. However, a recent study on GTP hydrolysis by the RhoA protein showed that the proton transfer required to recover the amide form of a Gln residue is rate-limiting.^12^ In the case of POFUT1, the catalytic mechanism leaves Asn51 in a tautomeric form that is thermodynamically disfavored under physiological conditions and whose persistence would be incompatible with continued catalysis. This implies that an active restoration step is required to reset the enzyme to its catalytically competent configuration, raising the mechanistic question of how Asn51 returns to its preferred amidic state (R–CONH_2_) before subsequent turnover (Figure 1A).

**Figure 1:**
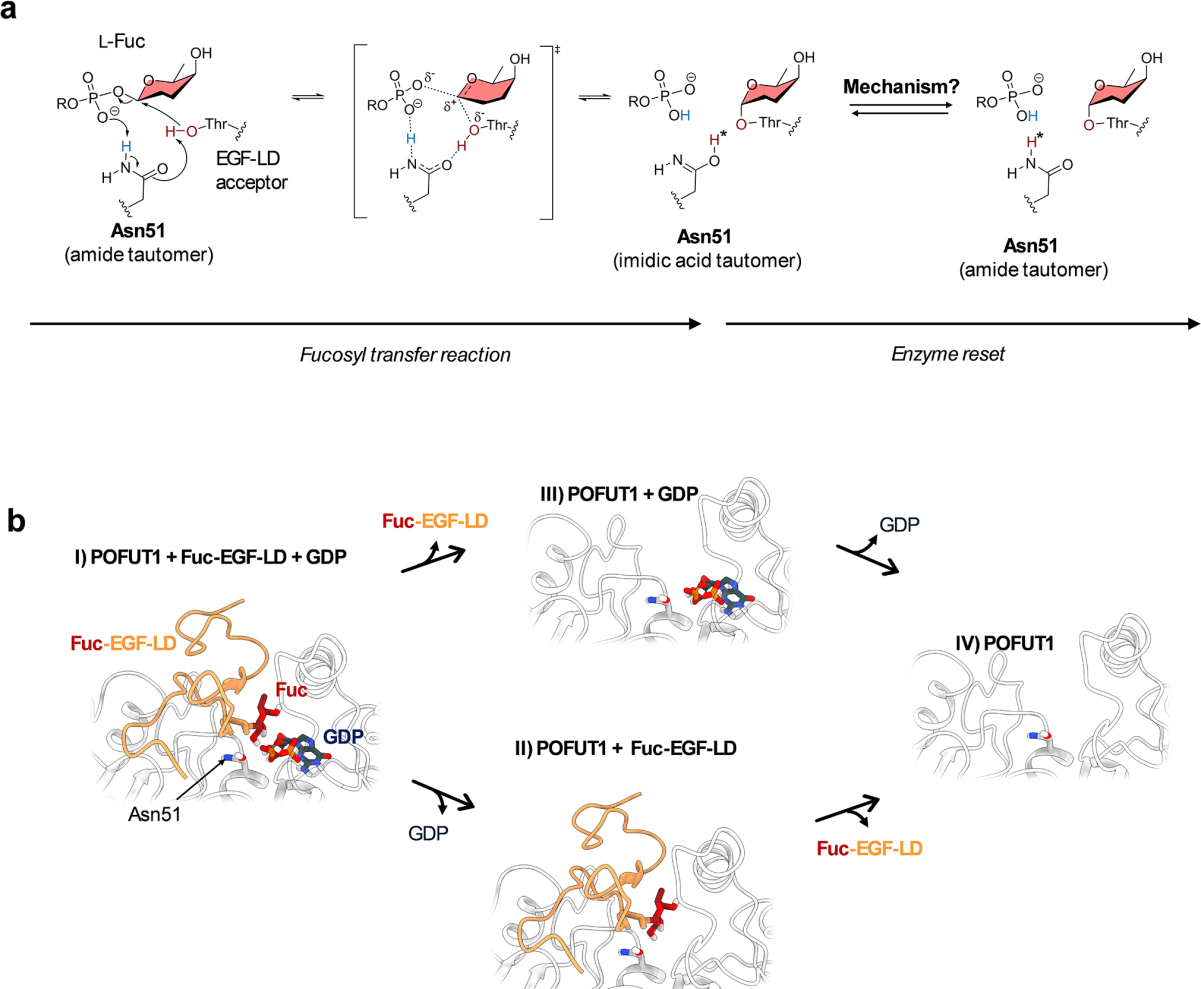
(a) Proposed enzymatic mechanism for *O*-fucosylation of the epidermal growth factor-like domain (EGF-LD) catalyzed by POFUT1. The reaction proceeds in a single step (S_N_2-type mechanism), leading to the formation of the imidic tautomer of the catalytic base Asn51. After the glycosyltransfer reaction, the enzyme resets by recovering the amide form of Asn51. The fucose ring is shown in salmon color, while atoms involved in bond cleavage and formation (hydroxyl group of the conserved threonine in the EGF-LD and the amine proton of Asn51) are highlighted in red and blue, respectively. (b) Plausible postcatalytic scenarios for the restoration of Asn51 to its amidic tautomer. From left to right: (I) immediately after the glycosyltransfer reaction, (II/III) after release of one of the reaction product molecules, and (IV) after release of the reaction products. The POFUT1 backbone is shown in gray, the glycosylated EGF-LD is shown in red/orange, and GDP is shown in dark blue.

The conversion between the amide/imidic acid forms of an amide functional group is expected to be mediated by water molecules or protein residues, as the intramolecular (direct) transfer of one proton involves a high-energy barrier.^13^ However, it remains an open question whether such proton transfer happens after the formation of the product complex (i.e., POFUT1 + GDP + fucosylated EGF-LD), once one of the product molecules (GDP or Fuc-EGF-LD) leaves the active site, or once both molecules have been released.

In the present study, we investigate the mechanism underlying the “tautomeric recovery” of the catalytic asparagine in POFUT1, i.e., how the hydroxyl proton (H* in Figure 1A) is transferred from the hydroxyl group to the imino group, in four possible postcatalytic scenarios (I–IV in Figure 1B). These are (I) the product complex (i.e., POFUT1 in complex with GDP and Fuc-EGF-LD); (II) the enzyme after release of GDP (i.e., POFUT1 + Fuc-EGF-LD); (III) the enzyme after release of the fucosylated peptide product (i.e., POFUT1 + GDP); and (IV) the enzyme after full product release (*i.e.*, unliganded POFUT1). By comparing the mechanism of proton shuttling in these four systems, we aim to identify the most likely scenario, the associated energetic cost, and the enzyme residues and water molecules involved in the tautomeric restoration of Asn51.

## 2 Results and Discussion

### 2.1 Postreaction Enzyme Dynamics

We performed 1 μs classical MD simulations to analyze the active site dynamics in each of the four investigated systems (I–IV). System stability, assessed by computing the root-mean-squared deviation (RMSD, Figure S1) of the protein backbone, showed differences among the four systems. The product complex (I) displayed the lowest average RMSD value, whereas the other systems showed broader distributions, with the unliganded enzyme (IV) exhibiting the largest structural deviation (Figures 2A and S2). This trend is consistent with the increase of solvent-accessible regions following the removal of the donor and/or acceptor molecules. Solvent-accessible surface area (SASA) calculations supported this observation, revealing a general increase in solvent exposure, particularly in the active site region (Figure 2B,C). These results suggest that FOFUT1 undergoes conformational rearrangements upon product release.

**Figure 2:**
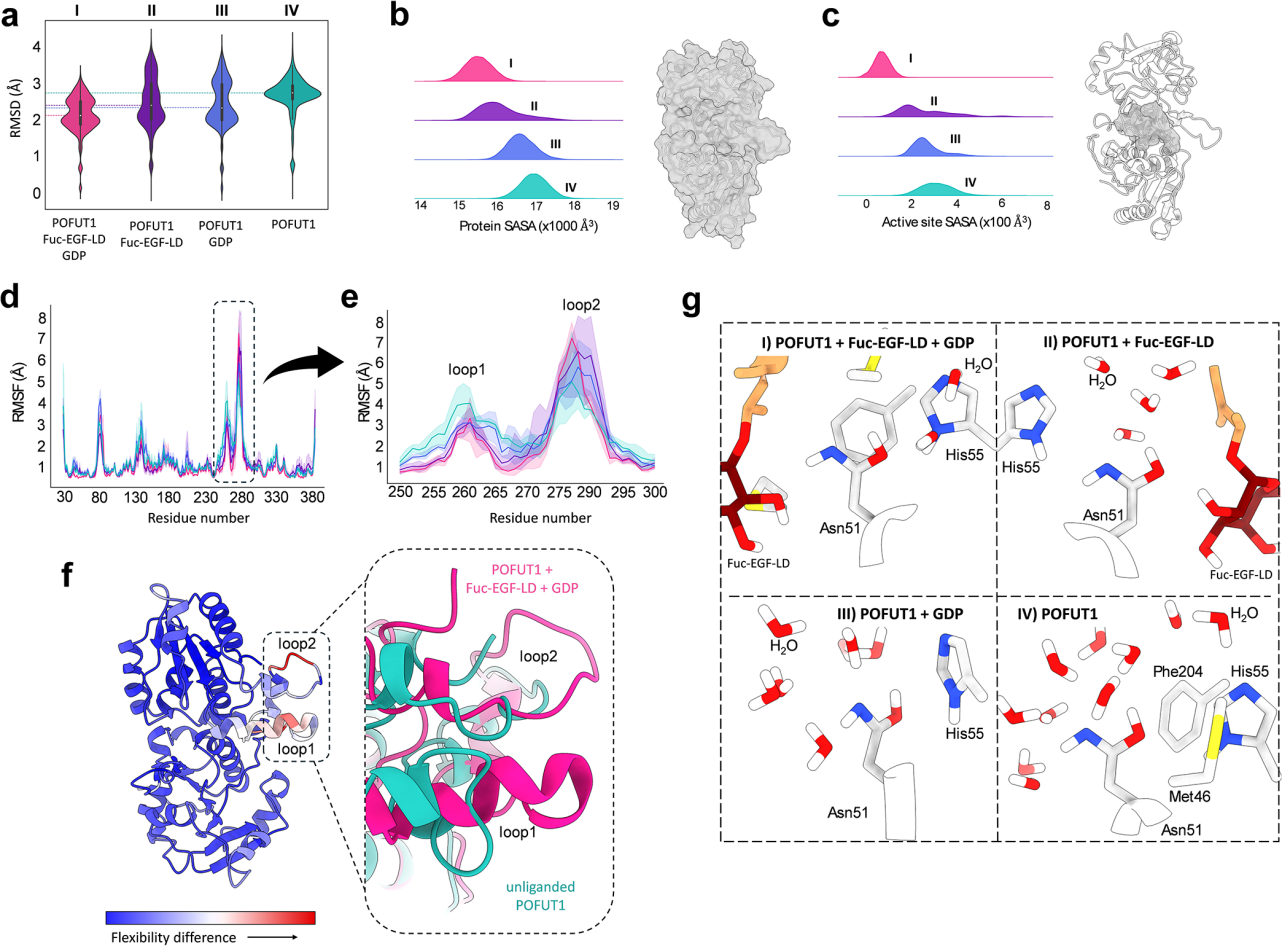
(a) Root-mean-squared deviation (RMSD) distributions of the POFUT1 backbone atoms along the simulations, referenced with respect to the product structure obtained from QM/MM simulations. The *y*-axis denotes RMSD values (Å), and the *x*-axis indicates the different systems studied. (b) Solvent-accessible surface area (SASA) distributions of the entire POFUT1 protein (surface representation in gray, right panel) for the various systems, shown in different colors. (c) SASA distributions computed for the active site residues (Met46, Gly47, Arg48, Gly50, Asn51, Gln52, His55, Phe204, Ser362), displayed as a gray surface on the right. (d) Root-mean-squared fluctuation (RMSF) analysis of Cα displacements in POFUT1 under different scenarios. (e) RMSF values for residues 250–300, corresponding to two flexible loop regions (termed loop 1 and loop 2). (f) Cartoon representation of the POFUT1 backbone flexibility, colored according to the RMSF difference between scenarios I and IV (blue: minimal; red: maximal). The inset highlights conformational changes in loop 1 and loop 2. (g) Local environment around the catalytic residue Asn51 in each scenario. Product release increases solvent exposure and thus the number of surrounding water molecules. Color coding for the four scenarios is consistent throughout the manuscript: I (magenta), II (purple), III (blue), and IV (green).

Protein flexibility, analyzed through root-mean-squared fluctuation (RMSF) calculations (Figures 2D and S3), identified two loops that change the most upon product release. Loop 1 (around residue 260) becomes more flexible, partially unfolding, whereas loop 2 (around residue 280) adopts a relatively rigid and compact conformation (Figures 2E,F and S4). These conformational adjustments after the catalytic cycle open the active site to accommodate donor and acceptor substrates for the next catalytic round.

To gain insight into possible pathways for the postcatalytic tautomerization of Asn51, we analyzed the local environment of the catalytic residue in the MD trajectories. Several protein residues are located in the vicinity of Asn51, including Met46, His55, and Phe204. However, none of them were found to form hydrogen-bonding interactions compatible with direct participation in proton transfer to or from the imidic group of Asn51. In particular, although His55 is the only nearby residue with potential acid–base character, its side chain is not oriented favorably toward the Asn51 heteroatoms. Therefore, neither protein side chains nor ligand fragments were found in suitable positions to assist with tautomerization. By contrast, the active site remains solvent-accessible, with water molecules occupying positions proximal to Asn51. This is particularly evident in the enzyme in complex with one product molecule or none (II–IV) and most prominently in the unliganded state (IV) (Figures 2G and S5). These observations suggest that the regeneration of Asn51 to its amide tautomer is mediated by water molecules through proton-exchange mechanisms rather than via protein residues.

### 2.2 Solvation of the Catalytic Asn Residue

In principle, successful Asn tautomerization will depend not only on the availability of water molecules in the active site but also on the probability of them having the appropriate orientation to allow proton shuttle between the imido and hydroxyl groups of the Asn side chain (Figure 3). Therefore, we analyzed the distribution of water molecules in the active site and their orientation across the four scenarios (I–IV).

**Figure 3:**
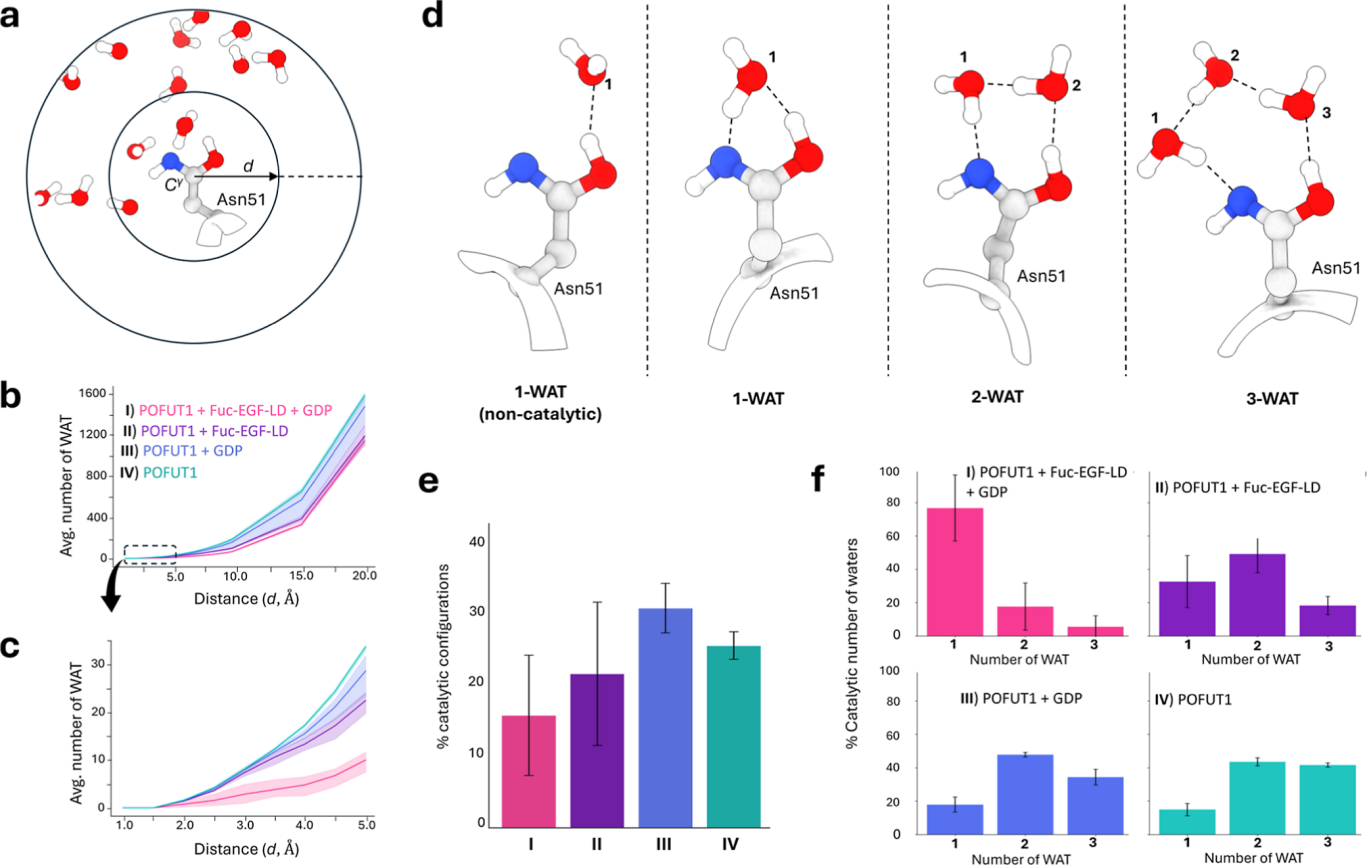
(a) Schematic representation of the strategy used to quantify the number of water molecules around the Asn51 side chain. Water molecules were counted within radial cutoffs ranging from 1 to 10 Å (0.5 Å increments) and additionally at 15 and 20 Å. (b) Average number of water molecules for each scenario, following the procedure described in panel a. (c) Enlarged view of the 1–5 Å region around Asn51, highlighting differences in the local environment of the catalytic residue across the different scenarios. (d) Representative conformations observed during the simulations, showing different arrangements of water molecules relative to the imidic acid group of Asn51. The residue can interact with water molecules either without forming a hydrogen-bond network (left) or through networks bridging the carbonyl and amine groups. The analysis was restricted to catalytically competent configurations mediated by up to three water molecules. (e) Average percentage of catalytically competent configurations observed in each scenario, with black error bars shown. (f) Percentage of catalytically competent configurations for all systems studied.

The solvation of the catalytic Asn51 residue was analyzed by quantifying the number of water molecules within increasing distances from the Cγ atom of the Asn51 side chain (Figure 3A). Water molecules were counted in shells from 1 to 10 at 0.5 Å increments as well as at 15 and 20 Å. At long distances, corresponding to bulk solvent, all systems converged to similar values (Figure 3B). In contrast, there are qualitative differences within the 1–5 Å region (Figure 3C). Whereas the binary complexes (scenarios II and III) and the unliganded enzyme (IV) show an increase in the number of water molecules as the distance increases, the ternary complex (I) shows little change. This indicates that the GDP and the fucosylated peptide (Fuc-EGF-LD) effectively shield the catalytic base Asn51 from the solvent, whereas dissociation of any of the two molecules enhances its solvation.

We next examined the formation of catalytically competent water networks around Asn51, which is a necessary condition for efficient proton migration. A catalytic configuration was defined as one in which both heteroatoms (nitrogen and oxygen) of the Asn side chain are engaged in hydrogen-bonding interactions either with the same water molecule (in a one-water-mediated tautomerization mechanism) or with two or three water molecules (Figure 3D). Our analysis revealed that the enzyme complex with GDP (III) exhibits the highest frequency of catalytically competent configurations, followed by the unliganded enzyme (IV), the enzyme complex with the fucosylated peptide (II), and finally the ternary complex (I) (Figure 3E). Notably, biochemical data showed that POFUT1/2 retain GDP with high affinity, indicating that the fucosylated product dissociates first.^9,14^ Our results suggest that the negative charge of the GDP phosphates attracts and facilitates the proper positioning of water molecules to enable the tautomeric transition. In fact, there is a predominance of two- and three-water-mediated configurations in systems lacking the Fuc-EGF-LD product (III and IV), whereas systems containing Fuc-EGF-LD (I and II) display a shift toward the one-water-mediated arrangement, particularly system I (Figure 3F). Clearly, the presence of the Fuc-EGF-LD product reduces the available space for water molecules to organize into suitable hydrogen-bond networks.

The involvement of water molecules observed here is consistent with previous computational studies highlighting the role of active site hydration and transient water networks in enzymatic catalysis. In particular, QM/MM and molecular dynamics investigations have shown that structured water molecules can facilitate proton transfer, stabilize reactive configurations, and lower free energy barriers through hydrogen-bond networks within the active site.^15,16^ In several enzymes, the dynamic organization of water molecules in active sites has been highlighted.^17,18^ In this context, our results extend these observations to POFUT1 by showing how the active site enables the formation of the competent configurations required for Asn51 tautomeric restoration.

### 2.3 Retautomerization of the Catalytic Asn

Since configurations involving two water molecules emerged as the predominant scenario, this case was selected to model the tautomerization reaction (Figure S6). For comparison, a one-water-mediated tautomerization was also evaluated. Representative structures containing either one or two water molecules bridging the two heteroatoms of the Asn51 side chain were extracted for each scenario I–IV (see Methods). In all cases, these water molecules remain solvent-exposed and do not interact with either the reaction products (GDP or Fuc-EGF-LD) or protein residues.

To investigate the tautomerization process, we performed a series of enhanced QM/MM simulations. First, we carried out exploratory simulations to promote tautomerization in the simplest scenario (the unliganded enzyme), using a chemically intuitive collective variable (CV) within the on-the-fly probability enhanced sampling (OPES)^14,15^ framework (see Methods for details). We then characterized in more detail all cases for the tautomerization process, corresponding to scenarios I–IV and considering both one- or two-water-mediated mechanisms in each case. To do so, we employed a novel enhanced sampling scheme based on a machine-learned committor,^16^ a robust method to improve the sampling of both reactive events and transition state (TS) configurations, which has recently been successfully applied to the study of enzymatic reactions.^17^ Using this protocol, we ran QM/MM-enhanced sampling simulations for the four systems I–IV, considering the two tautomerization scenarios (one- and two-water-mediated), yielding eight simulations in total (Figures S8–S11 and Supporting Information Table S1). We represented the resulting free energy profiles projected onto two chemically informative CVs (Figure 4) to facilitate their interpretation: the difference in distances involving the imide heteroatoms (CV1 = *d*_C–N_ – *d*_C–O_) and a difference in coordination numbers involving the imide group and water molecules (CV2; see methods for details). A shift from negative to positive CV1 values (from left to right on the FES) denotes the formation of the C–N single bond characteristic of the amidic tautomer. For CV2, values close to 0 correspond to states in which both nitrogen and oxygen atoms hold a proton each (imidic acid form), while higher values (CV2 ≈1.25 and 1.5) indicate configurations where the nitrogen atom holds two protons (amide form).

**Figure 4:**
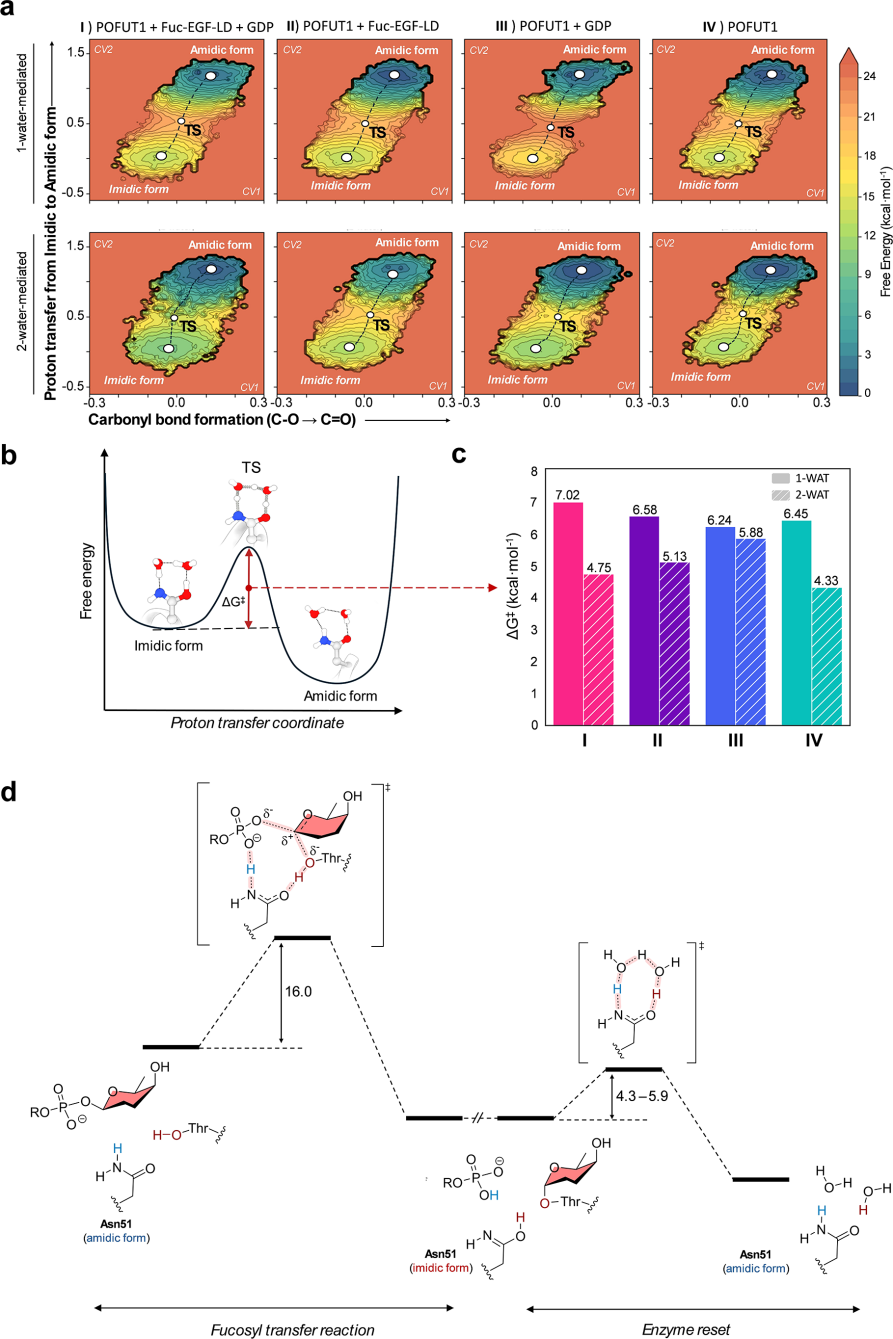
(a) Free energy landscapes of the tautomerization process mediated by one water molecule (top) and two water molecules (bottom) for scenarios I–IV (left to right). Progression along CV1 (left to right) indicates conversion of the C–O bond into a C=O bond, while movement along CV2 (bottom to top) corresponds to proton transfer, converting Asn from imidic acid to amide tautomer. (b) Schematic free energy profile of the two tautomeric forms of Asn51 in POFUT1. (c) Comparison of the computed tautomerization free energy barrier (Δ*G* ^‡^, in kcal·mol^–1^) in the four scenarios. Solid and dashed bars represent reactions mediated by one and two water molecules, respectively. (d) Energetic diagram integrating the fucosyltransfer reaction (left) and the enzyme reset process associated with the imidic-to-amidic tautomerization (right). Covalent bonds that are being broken or formed are indicated by pink shaded dotted lines. The discontinuity at the intermediate state represents a possible product release step. The reported range of activation barriers reflects the minimum and maximum values obtained for the two-water-mediated mechanism across the different scenarios analyzed in this study.

The results obtained show that in all cases, the amidic form of Asn51 is more stable (Figure 4A) than the imidic acid form by approximately 10 kcal·mol^–1^. This is similar to the values reported in prior theoretical studies on formamide/formamidic acid tautomerization in the gas phase or in small catalyst-assisted model systems, which lie between 11 and 13 kcal·mol^–1^.^13,19–23^ Therefore, the enzyme reset process leads to the most stable product complex. In all cases, the reaction proceeds in a concerted fashion, with a single TS separating the two stable states (amide and imidic acid forms of Asn51; Figures 4B, S12 and S13), with a free energy barrier (Δ*G* ^⧧^) in a range of 4–7 kcal·mol^–1^.

Despite this common picture, free energy barriers for the tautomerization process show quantitative differences (Figure 4C). The one-water-mediated mechanism consistently shows higher free energy barriers (Δ*G* ^⧧^ = 6.5–7.0 kcal·mol^–1^) than the two-water-mediated case (Δ*G* ^⧧^ = 4.3–5.9 kcal·mol^–1^). This reflects geometric constraints: a single water molecule cannot efficiently orient its O–H toward the N atom of the imidate group and, simultaneously, an oxygen atom lone pair toward the imidate hydroxyl group. In contrast, a two-water chain provides a higher flexibility and a more favorable arrangement around Asn51. A similar trend has been reported in previous potential energy calculations for the tautomerization of isolated formamidic acid to formamide: water assistance lowers the activation barrier, from ∼35–36 kcal·mol^–1^ in the gas phase to ∼10–11 and ∼8–9 kcal·mol^–1^ when one and two water molecules, respectively, participate in the proton transfer (Supporting Information Table S2).^13,24^

To gain further insight into the TS of each reaction, we clustered configurations sampled near the TS, selecting ten representative structures within ± *k*_B_ *T* of the highest energy region of the FES. In all systems, these TS configurations show that the proton from the OH group is being transferred to a water molecule, while the proton on the adjacent water molecule, located near the nitrogen atom, is simultaneously transferred (Figure 4B and Supporting Information Table S3). Distance analysis also shows that, at the TS, the C–N bond begins to elongate while the C–O bond begins to shorten. This trend is observed consistently in all scenarios, regardless of the number of water molecules involved. The main geometric differences between the one- and two-water-mediated mechanisms are observed in the angles. For instance, the < C–O–H and < C–N–H angles (where H is the hydrogen atom being transferred) are smaller in the one-water mechanism (e.g., 101.4 ± 2.4 and 106.4 ± 2.6 degrees, respectively, for scenario I) compared to the two-water mechanism (e.g., 118.1 ± 4.6 and 124.7 ± 1.9 degrees, respectively, for scenario I). This is consistent with the more constrained geometric arrangement of the water molecule in the one-water-mediated tautomerization mechanism. A single bridging water restricts the proton-transfer angle, yielding similar TS distances but distinct angular geometries and energetics (Figures 5, S12 and S13, and Supporting Information Table S3). Therefore, the two-water-mediated tautomerization mechanism appears to be the most likely, and this mechanism is operative (i.e., low free energy barrier) even if the reaction products have not yet been released from the active site.

**Figure 5:**
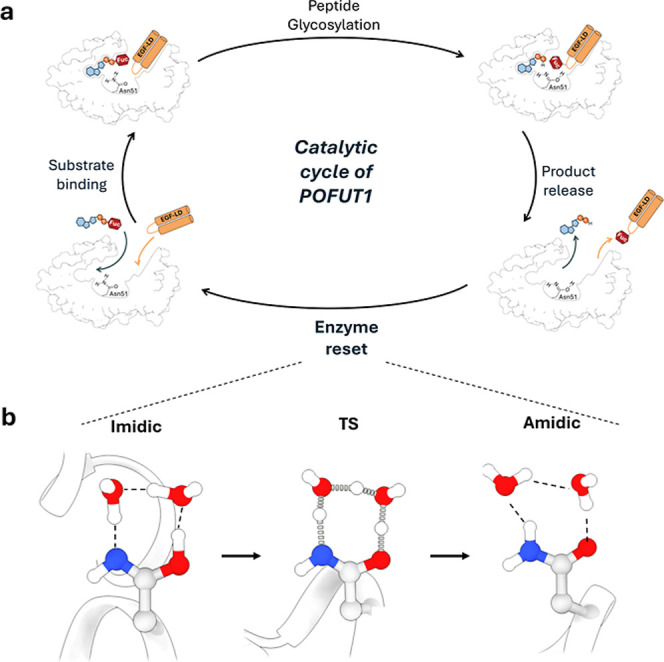
(a) Proposed catalytic cycle of POFUT1, in which the enzyme returns to its resting state after product release. (b) Tautomerization mechanism of POFUT1 mediated by two water molecules in the active site. At the TS, proton transfer occurs between the imidic acid side chain of Asn51 and the bridging water molecules. Distances and angles are reported in Table S2.

## 3 Conclusions

Our study shows that POFUT1 restores the canonical tautomeric state of its catalytic Asn51 residue through a water-mediated proton-transfer mechanism. The process proceeds via a Grotthuss-type relay and regenerates the most stable amidic form of Asn51. Across all postcatalytic scenarios examined, including systems with one or two bridging water molecules and with or without product molecules (GDP and Fuc-EGF-LD) in the active site, the computed free energy barriers remain low (Δ*G* ^⧧^ ≈4–7 kcal·mol^–1^). These barriers are substantially lower than those previously reported for the fucosyl transfer step (≈16 kcal·mol^–1^),^1^ indicating that tautomeric restoration is not rate limiting in the overall catalytic cycle. The most favorable case (Δ*G* ^⧧^ = 4.3 kcal·mol^–1^) corresponds to the two-water-mediated tautomerization after release of both reaction products from the active site (Figure 5). This value is also similar to those reported in prior theoretical studies on formamide tautomerization.^13,24^

Taken together, our results demonstrate that water molecules play an active catalytic role in restoring the resting state of the enzyme after turnover. More broadly, these findings highlight the importance of considering enzyme reset steps to achieve a complete mechanistic description of catalysis. Similar water-mediated strategies may also operate in other enzymes, in which catalytic residues undergo protonation state changes during turnover.

## 4 Methods

### 4.1 System Setup and Classical MD Simulations

The starting coordinates of the product complex were taken from a previous study of the fucosyl transfer mechanism.^11^ In that study, the Michaelis complex was assembled using crystal structures PDB 5KY3 and 5KXH^10^ and the reaction was modeled by QM/MM metadynamics. The computed structure of the product complex was used in this study. Four models (I–IV) in which tautomerization can take place were considered, corresponding to different postcatalytic scenarios: the full ternary complex (I), protein–Fuc-EGF-like-domain (II), protein–GDP (III), and unliganded protein (IV). Protonation states of titratable residues were assigned at pH 8.5 using MolProbity^25^ and PROPKA,^26^ in line with previous computational predictions. The Amber ff14SB force field^27^ was used for the protein, and GLYCAM06 ^28^ was used for carbohydrate residues. Atomic partial charges (RESP)^29^ were calculated for Asn51 in its imidic tautomeric form and the GDP moiety (overall charge −2) at the HF/6–31G* level of theory using Gaussian09.^30^ Parameters were generated with Antechamber.^31^ Topology and coordinate files were prepared in AmberTools23 (LEaP module).^32^ Each system was solvated in a rectangular water box with at least 10 Å of solvent on each side, and counterions were added for neutrality. Simulations were run in Amber24 ^33^ with the GPU-accelerated PMEMD module.

The energy minimization protocol consisted of three stages: (i) minimization of solvent, (ii) minimization of the protein with restraints, and (iii) minimization of the full system. Each stage included 2000 steps of steepest descent followed by up to 18,000 steps of conjugate gradient minimization. After minimization, the systems were gradually heated from 0 to 300 K in the *NVT* ensemble using the Berendsen thermostat (coupling constant 1.0 ps).^34^ This was followed by 200 ps of equilibration in the *NPT* ensemble at 300 K and 1 atm by using the Berendsen thermostat and barostat to stabilize the density. Production simulations were carried out in the *NPT* ensemble with a time step of 2 fs. The SHAKE algorithm^35^ was applied to all bonds involving hydrogen atoms. Temperature was controlled using the Langevin thermostat and pressure with the Berendsen barostat. Three independent replicas of 1 μs were run, yielding a total of 3 μs of simulation time for each system.

Trajectory analysis was performed using cpptraj (AmberTools23)^32^ and VMD^36^ to obtain RMSD and RMSF values. Solvent-accessible surface area (SASA) calculations were carried out using the surf command implemented in cpptraj, which estimates SASA using a rolling probe algorithm with a probe radius of 1.4 Å, corresponding to a water molecule. SASA was computed for each frame of the molecular dynamics trajectories and analyzed over the full simulation time for all systems. Two types of SASA analyses were performed: (i) the total SASA of the entire POFUT1 protein and (ii) the SASA of the active site, defined by residues Met46, Gly47, Arg48, Gly50, Asn51, Gln52, His55, Phe204, and Ser362. Distributions shown in Figure 2b,c therefore reflect the statistical sampling of solvent exposure across the complete MD trajectories, with differences in magnitude reporting on changes in the overall protein solvation and local active site accessibility, respectively. Water distributions around the catalytic Asn51 residue were quantified using MDTraj^37^ (compute_neighbors) with cutoffs from 1 to 10 Å (0.5 Å increments) and additionally at 15 and 20 Å. Catalytically competent water configurations were defined as conformations in which one or more water molecules form continuous hydrogen-bonded pathways capable of mediating proton transfer between the donor and acceptor heteroatoms of the Asn51 imidic group. These configurations were identified through hydrogen-bond network analysis using the MDAnalysis WaterBridgeAnalysis and HydrogenBondAnalysis tools (http://mdanalysis.org). Hydrogen bonds were defined using standard geometric criteria (donor–acceptor distance ≤3.5 Å and donor–hydrogen–acceptor angle ≥150°), and water bridges involving up to three water molecules were considered. The analysis was restricted to hydrogen bonds involving the heteroatoms of the Asn51 imidic group and water oxygen/hydrogen atoms. An in-house Python script was used to identify frames in which water molecules form continuous, directionally consistent hydrogen-bonded pathways connecting the donor and acceptor heteroatoms of Asn51, enforcing alternating donor–acceptor roles along the pathway, as required for a proton-shuttling mechanism.

### 4.2 QM/MM Simulations

Representative starting structures for the QM/MM MD simulations were selected by screening the full classical MD trajectories for frames containing catalytically competent configurations,^15,38^ as defined by the hydrogen-bond network criteria described above. For each scenario (I–IV), frames exhibiting either one- or two-water-mediated proton shuttling arrangements were identified, and a representative snapshot from each class was used to initiate the QM/MM simulations.^39^ For each system, two proton-transfer scenarios were considered: (i) mediated by one water molecule and (ii) mediated by two water molecules. This resulted in eight initial QM/MM simulations. The QM region included the side chain of Asn51 and the selected water molecules, keeping the QM region neutral and compact. QM/MM boundaries were treated using hydrogen link atoms within the IMOMM approach.^40^

Simulations were performed with CP2K (version 2024.3).^41^ The QM region was treated at the DFT level using the PBE functional^42^ and the Gaussian and plane-wave (GPW) formalism.^43^ The TZV2P basis set was used for the Gaussian expansion of the wave function, and an auxiliary plane-wave cutoff of 300 Ry was employed for the electron density. GTH pseudopotentials^44,45^ were applied to all atoms. The MM region was described with the Amber ff14SB force field.^27^ Each system was relaxed and equilibrated for 10 ps in the *NVT* ensemble with a coupling constant of 10 fs and a time step of 0.5 fs.

### 4.3 Enhanced Sampling Simulations

Enhanced sampling simulations were performed by applying two external bias potentials to the system. One is the OPES bias,^46^ employed to promote transitions between the two tautomeric states of the Asn51 side chain. The other is the Kolmogorov bias,^47^ aimed at favoring extensive sampling of the TS region, crucial for understanding the underlying catalytic mechanism. The latter bias is defined from an approximation of the committor function, which describes the probability of evolving to one state before the other. This function was parametrized and learned from data using a neural network (see Methods for details). From the learned committor function, a committor-based CV was derived and subsequently employed to connect reactants (Asn51 in the imidic acid form) and products (amide form), thereby driving the OPES bias. The following protocol was used:

a. Exploration of the tautomerization process using a physical collective variable (CV): using on-the-fly probability enhanced sampling (OPES) method and a linear combination of coordination numbers (CNs) as the CV, the tautomerization process was explored in the unliganded system for both one-water- and two-water-mediated mechanisms.
b. Training the committor-model: to have an accurate formal description of the reactive process, we trained a committor model using a neural network that takes physical descriptors of the system as inputs, based on the data obtained from the previous OPES simulations.
c. Enhanced the sampling of all systems using the committor function: the trained committor model was then employed as a CV, following the procedure described in ref 48 to enhance the sampling across all our systems (eight in total).

This approach provides a robust framework for improving the sampling of both reactive events and TS configurations. The three individual steps described above are described in more detail in the following sections.

#### 4.3.1 Exploration of the Tautomerization Process Using a Physical CV and OPES

The on-the-fly probability enhanced sampling method (OPES)^47,49^ was employed to accelerate sampling of the tautomerization mechanism. The biased CV was defined as the difference between two coordination numbers (CV = CN_1_ – CN_2_): CN_1_ is the sum of coordination numbers of each proton (except the initial NH proton) with the nitrogen atom of the Asn51 side chain, whereas CN_2_ is the sum of coordination numbers of each proton (except the initial NH proton) with the oxygen atom of the Asn51 side chain. Both coordination numbers were defined using rational switching functions, as described below

$${CN}_{1/2}=\sum_{i=1}^{n}\frac{{\left(1-\frac{r_{i}}{r_{0}}\right)}^{6}}{{\left(1-\frac{r_{i}}{r_{0}}\right)}^{12}}$$

Here, ***r***_***i***_ is the distance between the heteroatom and proton, while ***r***_0_ is the radial cutoff set to 1.1 Å. CNs were constructed from either five protons (*n* = 5, for two-water-mediated transfer) or three protons (*n* = 3, for a single-water-mediated transfer). Bias parameters were set as follows: a kernel deposition interval of 50 fs, a kernel bandwidth update every 100 fs, and an initial barrier height of 20 kcal·mol^–1^. This setup allowed accurate description of the TS while preventing exploration of noncatalytic high-energy regions. In the two-water-mediated case, simulations were run for 516 ps, yielding multiple TS crossings. For the single-water-mediated case, simulations were stopped after 81 ps, during which six TS crossings were observed.

#### 4.3.2 Committor Training and Enhanced Sampling

Following the procedure described in ref 48, we constructed a transferable, data-driven CV for the tautomerization reaction, where committor models were trained on configurations obtained from OPES exploration trajectories. The committor function *q*(*x*) was approximated using a neural network that takes as inputs some physical descriptors and combines them nonlinearly. Input descriptors were taken as distances between heteroatoms of catalytic water molecules and the Asn51 amidic acid group. Coordination numbers for hydrogen-bonded electronegative atoms are four in the two-water case and three in the single-water case.

The neural network used an architecture of [N, 32, 32, 1] nodes per layer with N descriptors as input (N = 9/13 for simulations using one/two bridging waters). Training employed the ADAM optimizer with an initial learning rate of 1 × 10^–3^, decayed exponentially with a factor γ = 0.9999. The α hyperparameter in the loss function was set to 1. Training was carried out for ∼50,000 epochs. A single iteration sufficed due to the extensive sampling obtained from OPES. Implementation was based on the open-source MLcolvar library.^46^

The trained committor CV, *z*(*x*), was used as a smoother reaction coordinate in subsequent enhanced sampling. The *z*(*x*) functions obtained from the one-water-mediated and two-water-mediated mechanisms served as transferable models used for the different scenarios studied in this work. Simulations coupled these CVs with both the Kolmogorov bias and standard OPES bias. Parameters included an energy barrier of 20 kcal·mol^–1^ and λ = 0.3. The resulting OPES and Kolmogorov bias potentials are shown in Figures S7–S11. Free energy profiles were projected onto two chemically informative CVs: the difference in distances involving the imide heteroatoms (CV1 = d_C–N_ – d_C–O_) and a difference in coordination numbers involving the imide group and water molecules (as described in the previous section “Exploration of the tautomerization process using a physical CV and OPES”). Configurations sampled near the highest energetic point along the *z*(*x*) function were clustered applying the k-medoid algorithm using the same descriptors employed in the training of the committor model.

#### 4.3.3 Advantages and Limitations of the Committor-Based Approach

It might be informative to discuss the advantages and limitations of the committor-based approach with respect to other popular methods, such as standard metadynamics based on a small number of geometric CVs. A key advantage of the committor-based approach is its possibility to encode complex reaction coordinates within a low-dimensional CV. Although formally one-dimensional, the committor can incorporate a large set of structural descriptors, thereby alleviating the dimensionality reduction problem commonly encountered when describing enzymatic reactions using a limited number of geometric parameters (distances, angles, etc.).

In the present implementation, the committor CV is coupled with OPES in addition to a Kolmogorov-type bias, which applies a minimal, targeted biasing potential designed to enhance sampling specifically in the TS region. This biasing strategy promotes sampling along the relevant reaction coordinate while largely preserving fluctuations in orthogonal degrees of freedom, thereby avoiding excessive distortion of the underlying free energy landscape. As a result, this approach is well suited to promoting transitions between metastable states while preserving the underlying physical fluctuations of the system.

The main limitation of the committor-based approach is the need for training data and, in some cases, iterative refinement of the CV. Constructing and validating a high-quality committor therefore require additional computational effort and a higher level of methodological expertise compared to standard metadynamics with predefined geometric CVs. While classical metadynamics remains a practical and robust choice for systems where suitable geometric CVs are readily available, the committor-based strategy is expected to be particularly advantageous for more complex enzymatic processes, where multiple coupled motions and solvent reorganization contribute to the reaction coordinate. In our case, it would have been tricky (but not impossible) to design a standard metadynamics simulation with CVs including all bond-breaking and bond-forming processes; therefore, we opted for the recently introduced ML-based committor approach.

## Supplementary Material



## Data Availability

Data files of the classical MD simulation and QM/MM OPES simulations generated in this study are deposited in Zenodo (10.5281/zenodo.17415036).

## Notes

Figures showing stability assessment of the systems, superimposition of representative snapshots before and after molecular dynamics (MD) simulations, the flexibility assessment of the systems, conformational changes observed in loops near the active site after molecular dynamics (MD) simulations, the solvent exposure of the active site, a schematic representation of the quantum mechanical/molecular mechanical (QM/MM) partition at the active site, the Kolmogorov bias along the collective variable (CV), CV evolution and OPES bias construction during the QM/MM of all scenarios, free energy profiles along the CV and along the minimum energy path extracted from the two-dimensional free energy surfaces, as well as tables with a summary of the QM/MM simulations, indicating computed activation barriers and the catalytic distances from the TS (PDF)

## Abbreviations

POFUT1: protein *O*-fucosyltransferase 1
EGF-LD: epidermal growth factor-like domains
NSP: Notch signaling pathway
GT: glycosyltransferase
GPW: Gaussian and plane-wave
OPES: on-the-fly probability enhanced sampling
CNs: coordination numbers
SASA: solvent-accessible surface area
RMSF: root-mean-squared fluctuation
TS: transition state.
