# Supplementary material for: Enzyme Reset: Water-Mediated Tautomerization Restores the Catalytic Asparagine in Protein O‑Fucosyltransferase 1
Source: J Chem Inf Model. 2026 Apr 10;66(10):6086–96. doi: 10.1021/acs.jcim.6c00350 (PMC13213826; doi:10.1021/acs.jcim.6c00350)
Supplement: Supplementary file 1 [file ci6c00350_si_001.pdf]

Supporting Information

Enzyme Reset: Water-Mediated Tautomerization Restores the Catalytic Asparagine in Protein *O*-Fucosyltransferase 1

Òscar Vidal-Gironès[1], Enrico Trizio[2], Peilin Kang[2], Michele Parrinello[2],

José Pablo Rivas-Fernández[1,], Carme Rovira[1,3*]*

[1]Departament de Química Inorgànica i Orgànica (Secció de Química Orgànica) and Institut de Química Teòrica i Computacional (IQTCUB), Universitat de Barcelona, Martí i Franquès 1, 08028 Barcelona, Spain

[2]Atomistic Simulations, Istituto Italiano di Tecnologia, Via Enrico Melen 83, 16142 Genoa, Italy

[3]Institució Catalana de Recerca i Estudis Avançats (ICREA). Passeig Lluís Companys 23, 08010 Barcelona, Spain

* Corresponding authors' e-mail addresses: jprivas@ub.edu, c.rovira@ub.edu

Contents

Figure S1. Stability assessment of the systems during molecular dynamics (MD) simulations (RMSD analysis for scenarios I–IV).

Figure S2. Superimposition of representative structures before and after MD simulations.

Figure S3. Flexibility assessment of the systems during MD simulations (RMSF analysis of POFUT1 Cα atoms).

Figure S4. Conformational changes observed in loops near the active site after MD simulations.

Figure S5. Solvent exposure of the active site after MD simulations.

Figure S6. Schematic representation of the quantum mechanical/molecular mechanical (QM/MM) partition at the active site.

Figure S7. Kolmogorov bias applied along the collective variable used in the QM/MM simulations.

Figure S8. Committor CV evolution and OPES bias construction during QM/MM simulations of scenario I.

Figure S9. Committor CV evolution and OPES bias construction during QM/MM simulations of scenario II.

Figure S10. Committor CV evolution and OPES bias construction during QM/MM simulations of scenario III.

Figure S11. Committor CV evolution and OPES bias construction during QM/MM simulations of scenario IV.

Figure S12. Computed free energy profiles along the committor CV for the studied mechanisms.

Figure S13. Free energy profiles along the minimum energy path extracted from the two-dimensional free energy surfaces.

Table S1. Summary of QM/MM simulations including simulation times and computed activation barriers.

Table S2. Catalytic distances and angles extracted from TS configurations obtained in the QM/MM simulations.

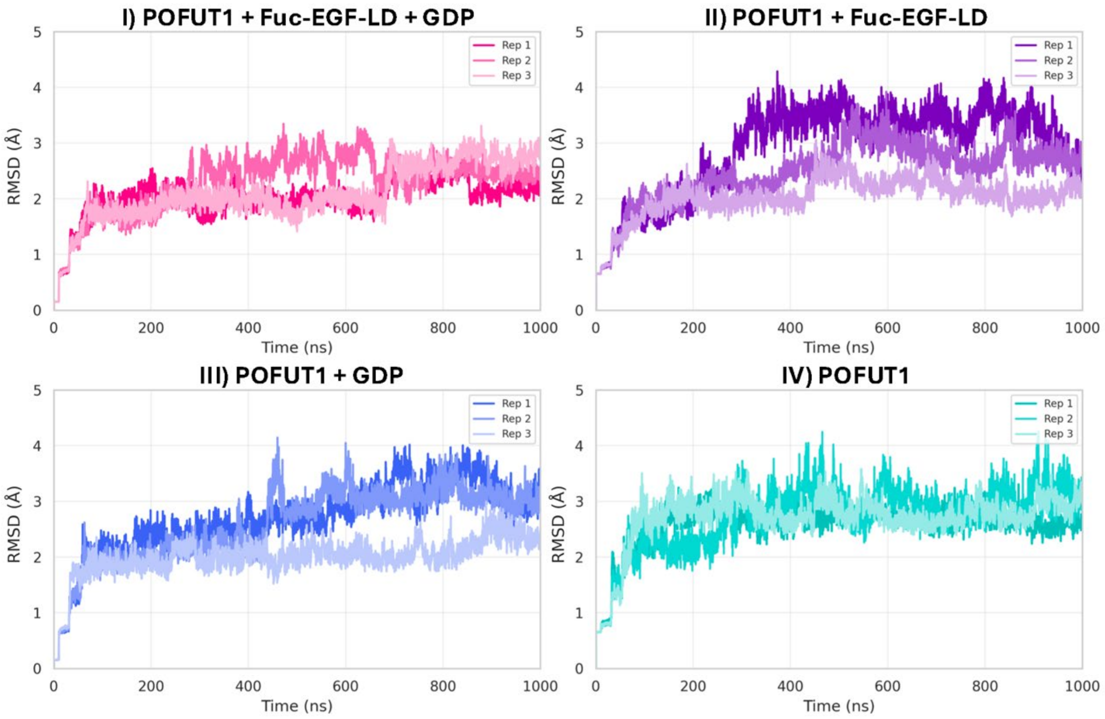

Figure S1. Stability assessment of the systems during molecular dynamics (MD) simulations. Root-mean-square deviation (RMSD) plots for the four studied scenarios along their respective trajectories. The top left, top right, bottom left, and bottom right panels correspond to scenarios I–IV, respectively. The y-axis represents RMSD values (Å), and the x-axis indicates simulation time (ns). Each graph shows data from triplicate simulations, represented by different colors.

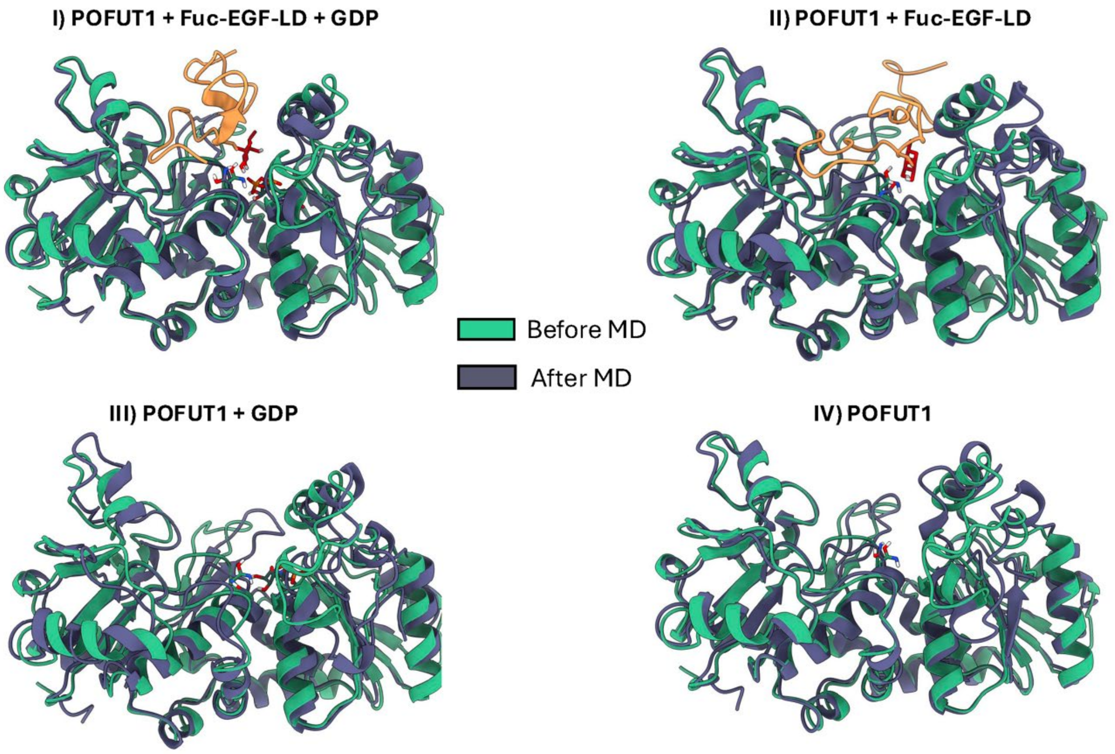

Figure S2. Superimposition of representative snapshots before and after molecular dynamics (MD) simulations. The top left, top right, bottom left, and bottom right panels correspond to scenarios I–IV, respectively. The cartoon in green color represents the conformation prior to MD, while the grey color denotes the structure obtained at the end of the MD simulation. The glycosylated EGF-like domain is shown in orange, and the sugar unit, catalytic Asn51 residue, and GDP moiety are displayed in stick representation.

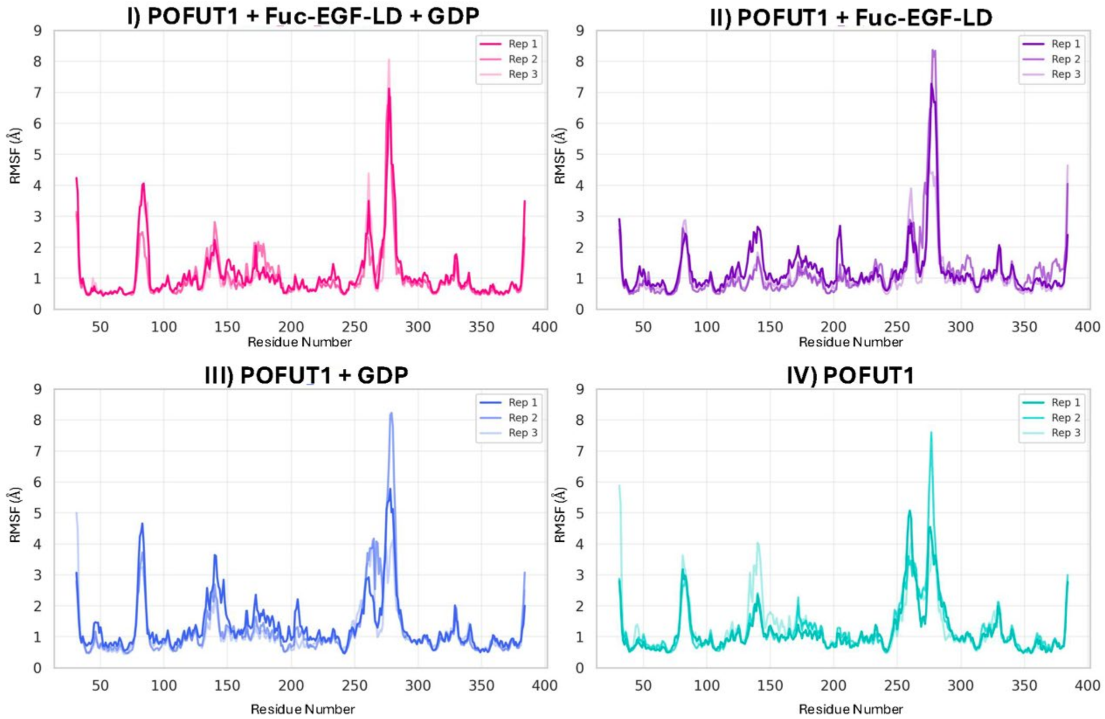

Figure S3. Flexibility assessment of the systems during molecular dynamics (MD) simulations. Root-mean-square fluctuation (RMSF) values obtained from the Cα atoms of the POFUT1 enzyme in the four studied scenarios. The top left, top right, bottom left, and bottom right panels correspond to scenarios I–IV, respectively. The y-axis represents RMSF values (Å), while the x-axis indicates enzymatic residues (labelled from 30 to 384). Each graph shows data from triplicate simulations, represented by different colors.

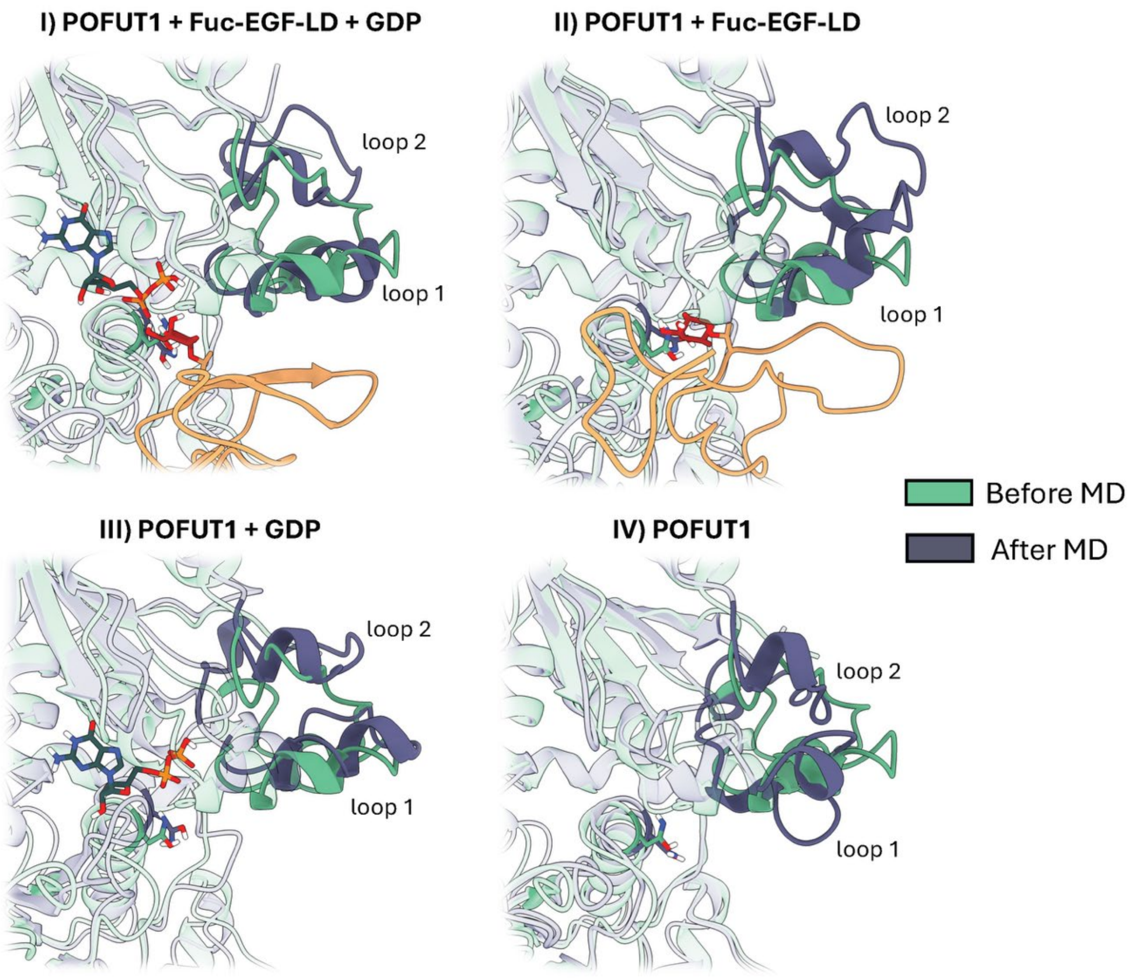

Figure S4. Conformational changes observed in loops near the active site after molecular dynamics (MD) simulations. Close-up views of the regions near the active site, corresponding to loop-1 and loop-2 (highlighted with arrows), showing the superimposition of structures before and after MD simulations. The top left, top right, bottom left, and bottom right panels correspond to scenarios I–IV, respectively. The cartoon in green color represents the conformation prior to MD, while the grey color denotes the structure obtained at the end of the MD simulation. The glycosylated EGF-like domain is shown in orange, and the sugar unit, catalytic Asn51 residue, and GDP moiety are displayed in stick representation.

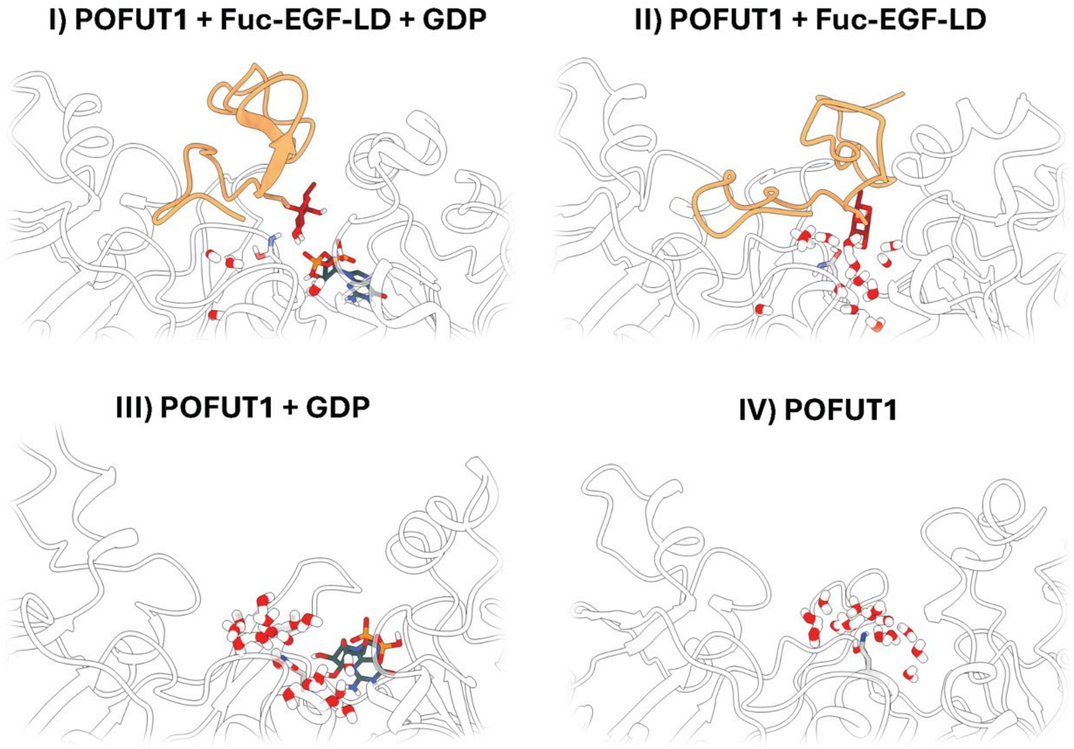

Figure S5. Solvent exposure of the active site after molecular dynamics (MD) simulations. The presence of ligands, particularly the EGF-like domain, clearly reduces the accessibility of water molecules to the catalytic site. The top left, top right, bottom left, and bottom right panels correspond to scenarios I–IV, respectively. The glycosylated EGF-like domain is shown in orange, and the sugar unit, catalytic Asn51 residue, GDP moiety, and water molecules are displayed in stick representation.

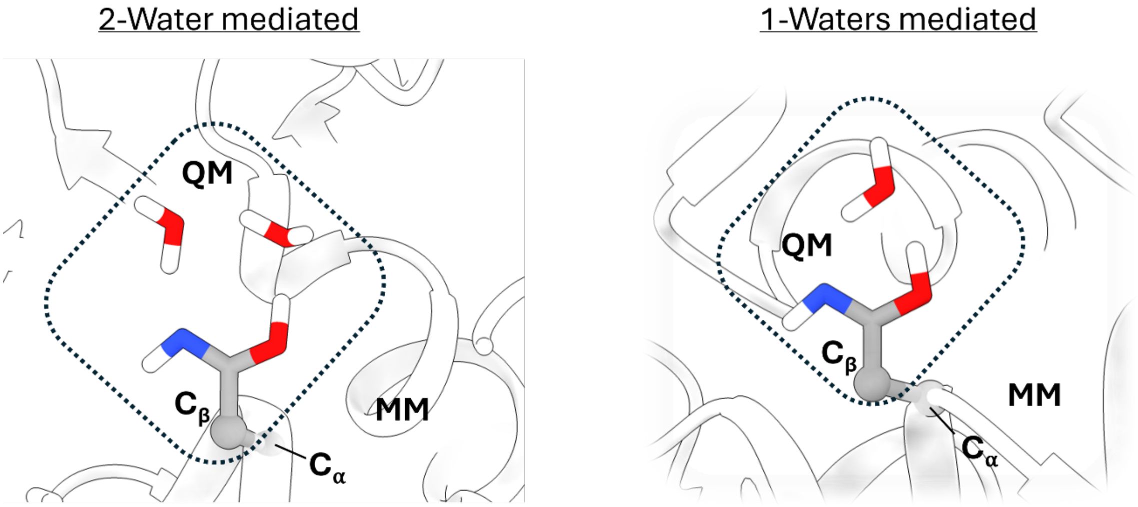

Figure S6. Schematic representation of the quantum mechanical/molecular mechanical (QM/MM) partition at the active site. The QM region comprises the side chain of the Asn51 residue (grey and colored portion of the stick representation) together with the atoms of the water molecules involved in the tautomerization process—six atoms (two water molecules) or three atoms (one water molecule) corresponding to the two-water- or one-water-mediated mechanisms, respectively. The remainder of the system was treated at the MM level, with a single QM/MM boundary defined between the Cβ (QM) and Cα (MM) atoms of the Asn51 residue (highlighted with spheres).

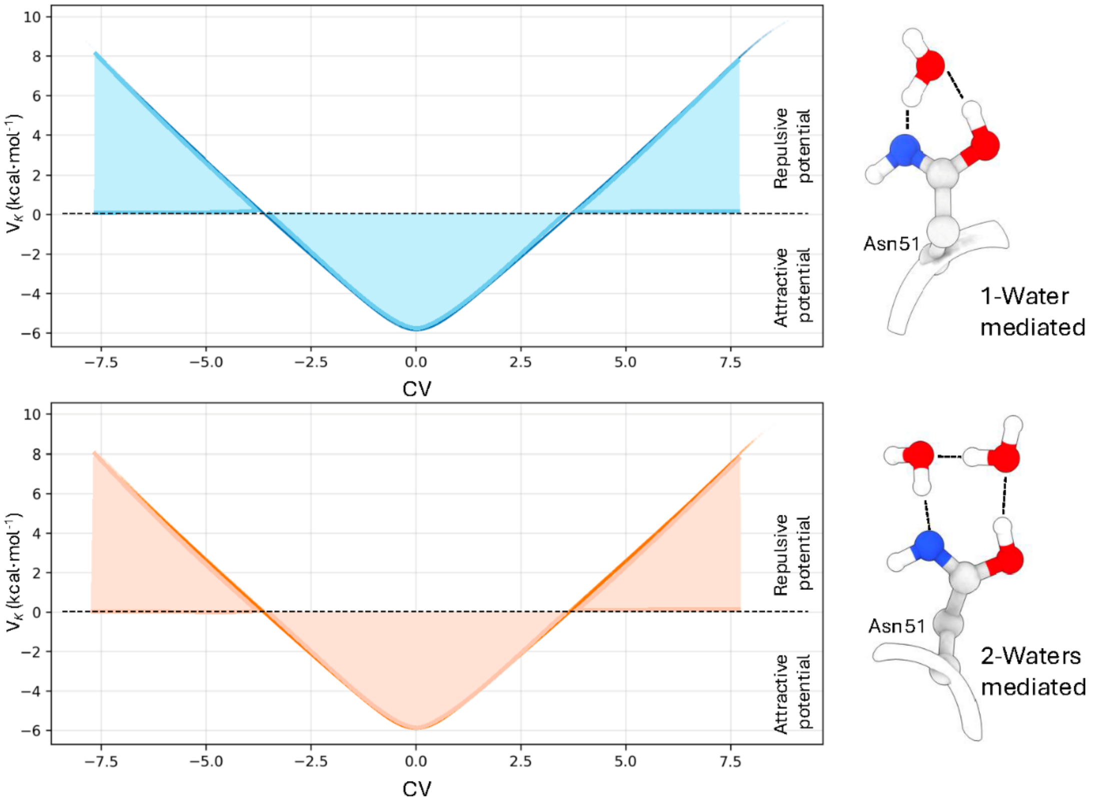

Figure S7. Kolmogorov bias along the collective variable (CV) used in the QM/MM simulations. The top panel shows the external Kolmogorov potential applied to the one-water-mediated simulations (blue), and the bottom panel corresponds to the two-water-mediated simulations (orange). The y-axis represents the potential value, while the x-axis indicates the CV. The potential depends solely on the mechanism studied, as the respective CV model (one- or two-water-mediated) was transferred across the four Scenarios. The CV is dimensionless, and the Kolmogorov bias potential is expressed in kcal·mol^{-1}.

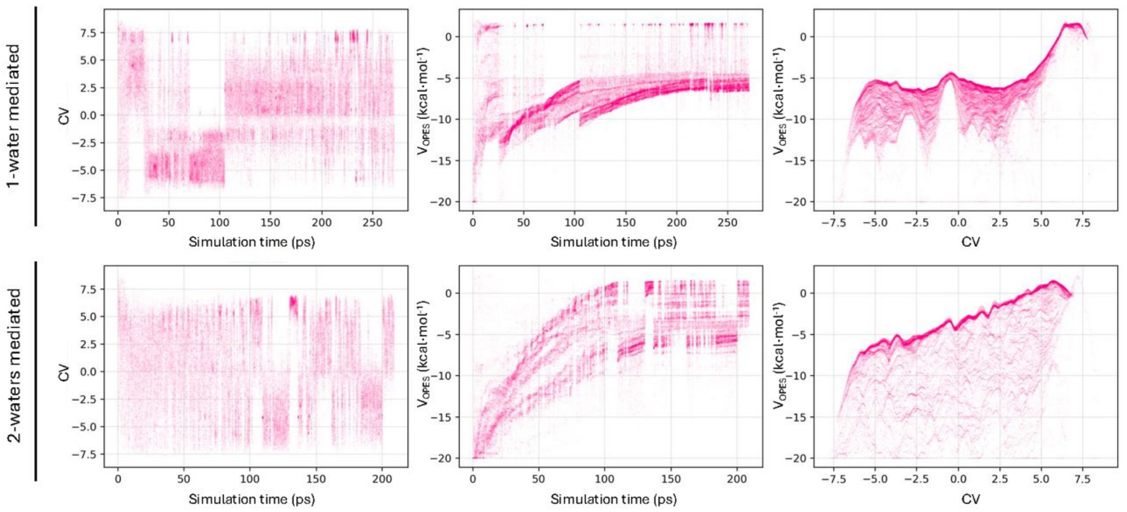

Figure S8. Collective variable (CV) evolution and on-the-fly probability-enhanced sampling (OPES) bias construction during the QM/MM simulations of scenario I. The top panels correspond to the one-water-mediated mechanism, while the bottom panels depict the two-water-mediated mechanism. The plots on the left show the evolution of the CV as a function of simulation time, whereas the middle and right plots display the evolution of the OPES bias with time and the bias construction along the CV, respectively. The CV is dimensionless, simulation time is given in picoseconds (ps), and the bias potential is expressed in kcal·mol^{-1}.

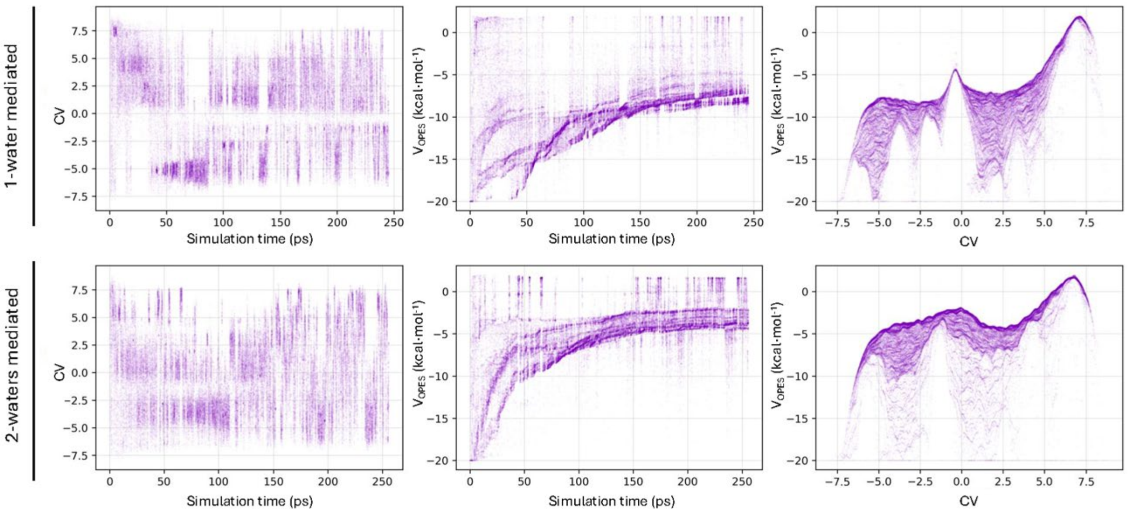

Figure S9. Collective variable (CV) evolution and OPES bias construction during the QM/MM simulations of scenario II. The top panels correspond to the one-water-mediated mechanism, while the bottom panels depict the two-water-mediated mechanism. The plots on the left show the evolution of the CV as a function of simulation time, whereas the middle and right plots display the evolution of the OPES bias with time and the bias construction along the CV, respectively. The CV is dimensionless, simulation time is given in picoseconds (ps), and the bias potential is expressed in kcal·mol^{-1}.

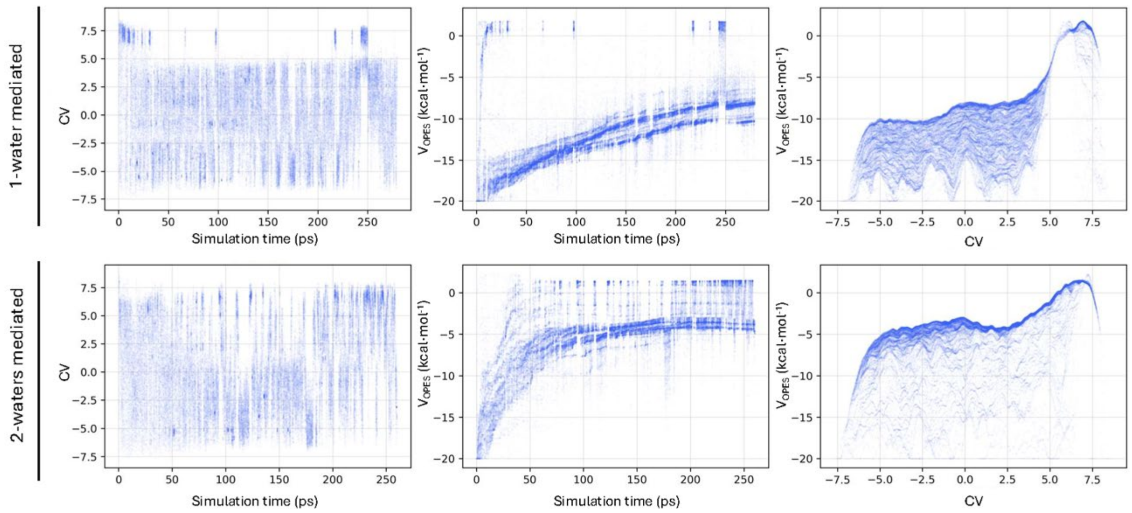

Figure S10. Collective variable (CV) evolution and OPES bias construction during the QM/MM simulations of scenario III. The top panels correspond to the one-water-mediated mechanism, while the bottom panels depict the two-water-mediated mechanism. The plots on the left show the evolution of the CV as a function of simulation time, whereas the middle and right plots display the evolution of the OPES bias with time and the bias construction along the CV, respectively. The CV is dimensionless, simulation time is given in picoseconds (ps), and the bias potential is expressed in kcal·mol^{-1}.

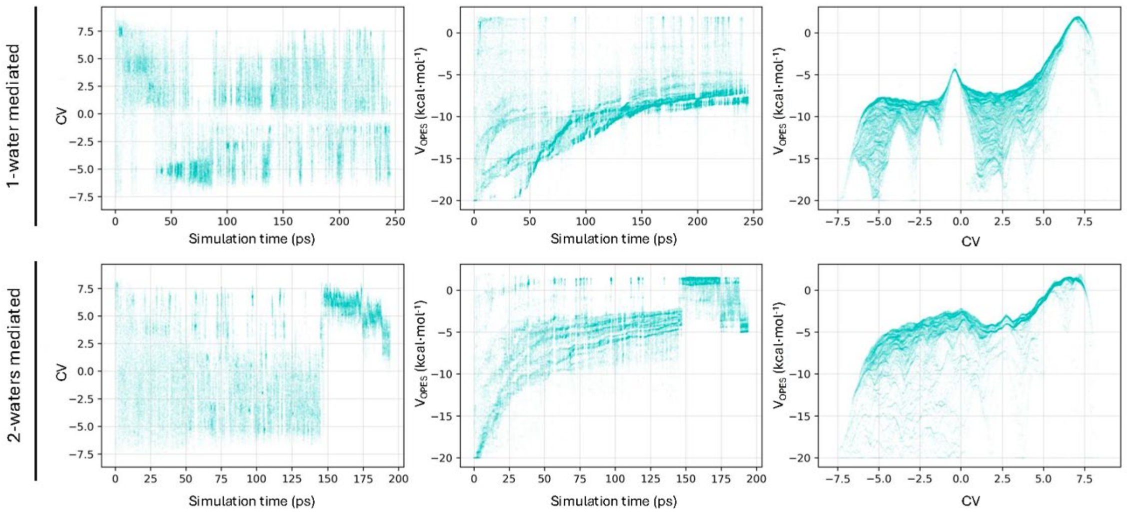

Figure S11. Collective variable (CV) evolution and OPES bias construction during the QM/MM simulations of scenario IV. The top panels correspond to the one-water-mediated mechanism, while the bottom panels depict the two-water-mediated mechanism. The plots on the left show the evolution of the CV as a function of simulation time, whereas the middle and right plots display the evolution of the OPES bias with time and the bias construction along the CV, respectively. The CV is dimensionless, simulation time is given in picoseconds (ps), and the bias potential is expressed in kcal·mol^{-1}.

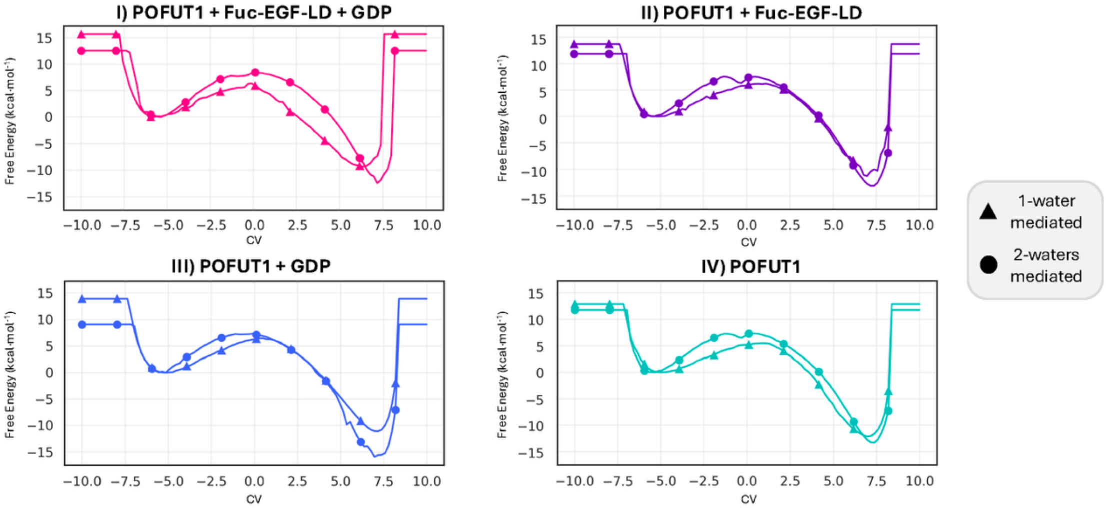

Figure S12. Computed free energy profiles along the collective variable (CV) used in the QM/MM simulations. Profiles represented with circles correspond to the one-water-mediated mechanism, while profiles with triangles correspond to the two-water-mediated mechanism. The top left, top right, bottom left, and bottom right panels correspond to scenarios I–IV, respectively. The y-axis represents the free energy (kcal·mol^{-1}), and the x-axis indicates the CV, which is dimensionless.

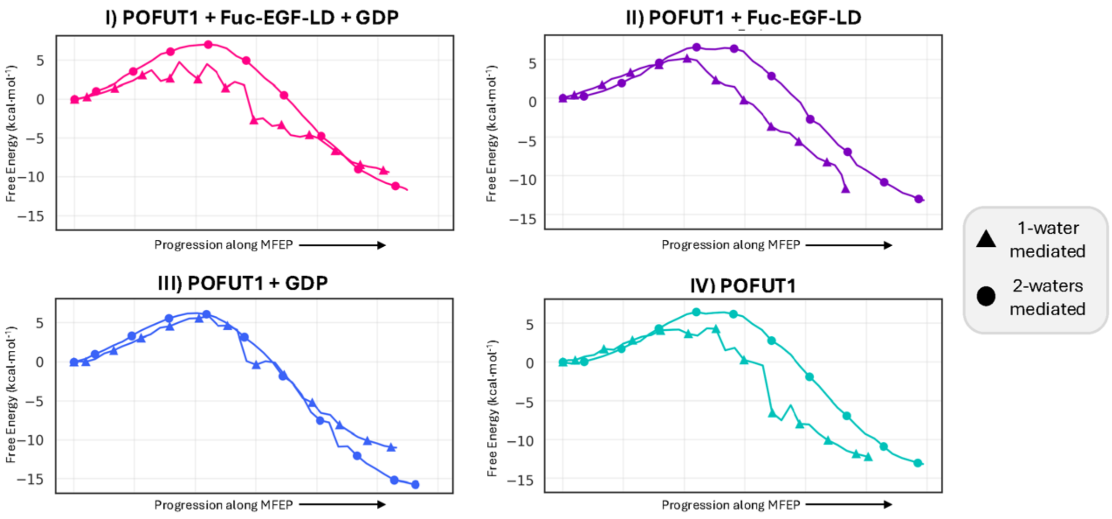

Figure S13. Free energy profiles along the minimum energy path extracted from the two-dimensional free energy surfaces. Profiles represented with circles correspond to the one-water-mediated mechanism, while profiles with triangles correspond to the two-water-mediated mechanism. The top left, top right, bottom left, and bottom right panels correspond to scenarios I–IV, respectively. The y-axis represents the free energy (kcal·mol^{-1}), and the x-axis indicates the collective variable (CV), which is dimensionless.

Supplementary Table 1. Summary of the QM/MM simulations, indicating the simulation time for each run and the computed activation barriers ($\Delta G^{\ddagger}$). Simulation time is reported in picoseconds (ps), and energies are expressed in kcal·mol^{-1}.

Scenario	# bridging waters	Total simulation time	ΔG‡
I	1	271.1	7.02
I	2	209.1	4.75
II	1	245.4	6.58
II	2	255.9	5.13
III	1	279.4	6.24
III	2	260.1	5.88
IV	1	245.4	6.45
IV	2	194.1	4.33

Supplementary Table 2. Summary of computed activation barriers in this work compared with prior theoretical studies in small models. The level of theory for the literature values is B3LYP/6-311++G(2df,2pd). The energy barriers in Du et al. [1](not reported in that study) were estimated for imidic to amidic direction by utilizing the thermodynamic energy difference between the imidic and amidic form using the B3LYP/6-311++G(2df,2pd) level of theory reported by Constantino *et al.*[2,1]

Reference	Water Molecules	Activation Barrier (kcal·mol^{-1})
Constantino et al., 2003	0	35.5
	1	11.4
Du et al., 2004	0	~36.0
	1	~10.1
	2	~8.9
POFUT1 (present work)	1	6.24 - 7.02
	2	4.33 - 5.88

Supplementary Table 3. Relevant distances and angles extracted from the transition-state (TS) configurations obtained in the QM/MM simulations. Distances are given in angstroms (Å) and angles in degrees (°). Statistics were derived from representative structures of n = 10 clusters per state, weighted according to the number of structures they represent.

scenario	waters	C-O	C-N	O-H	N-H	< C-O-H	<C-N-H
I	1	1.30 ± 0.02	1.33 ± 0.01	1.30 ± 0.04	1.38 ± 0.08	101.4 ± 2.4	106.4 ± 2.6
I	2	1.29 ± 0.04	1.33 ± 0.03	1.28 ± 0.04	1.43 ± 0.18	118.1 ± 4.6	124.7 ± 1.9
II	1	1.31 ± 0.01	1.32 ± 0.02	1.31 ± 0.01	1.40 ± 0.08	103.7 ± 2.2	105.1 ± 2.8
II	2	1.29 ± 0.01	1.31 ± 0.02	1.35 ± 0.04	1.50 ± 0.10	116.4 ± 2.3	125.2 ± 3.1
III	1	1.31 ± 0.01	1.31 ± 0.01	1.25 ± 0.05	1.41 ± 0.05	103.1 ± 3.3	105.1 ± 2.5
III	2	1.29 ± 0.02	1.32 ± 0.02	1.28 ± 0.02	1..48 ± 0.14	115.2 ± 3.6	125.6 ± 3.4
IV	1	1.30 ± 0.02	1.32 ± 0.02	1.29 ± 0.03	1.41 ± 0.11	104.7 ± 2.5	106.7 ± 1.6
IV	2	1.29 ± 0.02	1.32 ± 0.02	1.28 ± 0.04	1.48 ± 0.05	115.2 ± 3.7	125.6 ± 2.8

References

(1) Du, D.; Fu, A.; Zhou, Z. Density functional theory study of formamide–formamidic acid tautomerization. *Int. J. Quant. Chem.* **2004**, *99*, 1-10.

(2) Constantino, E.; Solans-Monfort, X.; Sodupe, M.; Bertran, J. Basic and acidic bifunctional catalysis: application to the tautomeric equilibrium of formamide. *Chem. Phys.* **2003**, *295*, 151-158.
